# Determination of ammonia and hydrogen sulfide emissions from a commercial dairy farm with an exercise yard and the health-related impact for residents

**DOI:** 10.1007/s11356-020-09858-y

**Published:** 2020-06-30

**Authors:** Chuandong Wu, Fan Yang, Marlon Brancher, Jiemin Liu, Chen Qu, Martin Piringer, Günther Schauberger

**Affiliations:** 1 School of Chemistry and Biological Engineering, University of Science and Technology Beijing, Beijing, 100083 China; 2 Beijing Municipal Research Institute of Environmental Protection, Beijing, 100037 China; 3 WG Environmental Health, Unit for Physiology and Biophysics, University of Veterinary Medicine, Vienna, Austria; 4 Department of Environmental Meteorology, Central Institute of Meteorology and Geodynamics, Vienna, Austria

**Keywords:** Emission, Ammonia, Hydrogen sulfide, Health risk, Dispersion model, Dairy farm

## Abstract

Airborne emissions from concentrated animal feeding operations (CAFOs) have the potential to pose a risk to human health and the environment. Here, we present an assessment of the emission, dispersion, and health-related impact of ammonia and hydrogen sulfide emitted from a 300-head, full-scale dairy farm with an exercise yard in Beijing, China. By monitoring the referred gas emissions with a dynamic flux chamber for seven consecutive days, we examined their emission rates. An annual hourly emission time series was constructed on the basis of the measured emission rates and a release modification model. The health risk of ammonia and hydrogen sulfide emissions around the dairy farm was then determined using atmospheric dispersion modeling and exposure risk assessment. The body mass-related mean emission factors of ammonia and hydrogen sulfide were 2.13 kg a^−1^ AU^−1^ and 24.9 g a^−1^ AU^−1^, respectively (one animal unit (AU) is equivalent to 500 kg body mass). A log-normal distribution fitted well to ammonia emission rates. Contour lines of predicted hourly mean concentrations of ammonia and hydrogen sulfide were mainly driven by the meteorological conditions. The concentrations of ammonia and hydrogen sulfide at the fence line were below 10 μg m^−3^ and 0.04 μg m^−3^, respectively, and were 2–3 orders of magnitude lower than the current Chinese air quality standards for such pollutants. Moreover, the cumulative non-carcinogenic risks (HI) of ammonia and hydrogen sulfide were 4 orders of magnitudes lower than the acceptable risk levels (HI = 1). Considering a health risk criterion of 1E-4, the maximum distance from the farm fence line to meet this criterion was nearly 1000 m towards north-northeast. The encompassed area of the contour lines of the ambient concentration of ammonia is much larger than that of hydrogen sulfide. However, the contour lines of the ammonia health risk are analogous to those of hydrogen sulfide. In general, the ammonia and hydrogen sulfide emissions from the dairy farm are unlikely to cause any health risks for the population living in the neighborhood.

## Background

Concentrated animal feeding operations (CAFOs), such as dairy and cattle farms, have been extensively developed in recent decades to meet peoples’ demands for meat and dairy products (Hu et al. 2017). Gaseous compounds emitted from dairy farms have evoked increasing social and environmental concerns (Jahne et al. 2015; Wang et al. 2018). Accordingly, residents are concerned with potential health risks due to gas emissions from dairy farms. The airborne emissions from dairy feedlots mainly consist of greenhouse gases (methane, nitrous oxides, and carbon dioxide) and pollutant gases (ammonia (NH_3_), hydrogen sulfide (H_2_S), volatile fatty acids (VFAs), phenols, and others) (Hales et al. 2015; Hales et al. 2012; Lee et al. 2018; Wang et al. 2018). Previous studies have shown that livestock production contributes extensively to NH_3_ and H_2_S emissions (Feilberg et al. 2017; Maasikmets et al. 2015).

Emissions of noxious gases such as NH_3_ and H_2_S could be worrying due to their malodorous and hazardous properties and are responsible for the acidification of ecosystems and the formation of secondary particulate matter (Maasikmets et al. 2018). Workers and neighbors of CAFOs such as dairy farms can be directly exposed to emitted noxious gases primarily through inhalation. Long-term exposure to these pollutants has been associated with potential health risks such as respiratory irritation and central nervous system damage (Jaars et al. 2018; Wu et al. 2018). Thus, there is a need to evaluate the potential health risks induced by noxious gases emitted from CAFOs such as dairy farms.

Several methods exist for the determination of gas emissions from CAFOs (Liu et al. 2017). Dynamic flux chamber is a direct measurement method. It has been widely used for the calculation of gas emission rates from passive surface sources (Parker et al. 2013b). Such measurements of the emission rates are needed, for instance, to estimate ambient concentrations at receptor points surrounding the emission source using dispersion models (Brancher et al. 2017; Schauberger et al. 2012). However, limited systematic studies assessing the ambient concentrations and the related health risk caused by noxious gas emissions from dairy farms can be found in the literature. Several of them have focused on odor, NH_3_, VFAs, and phenols (Hales et al. 2012; Lee et al. 2018; Rørvang et al. 2017), but H_2_S emissions can be more odorous and toxic due to its lower odor threshold value and reference concentration (RfC) value for chronic inhalation exposure. Studies on the assessment of noxious gas emissions from dairy farms with a large exercise yard are still limited (Keck 1997). Moreover, the related studies were mainly conducted in developed countries, whereas the dispersion and risks of NH_3_ and H_2_S emitted from dairy farms in China have not yet been well studied.

In this study, we present an assessment of the emission and the related human health risks of NH_3_ and H_2_S from a dairy farm in Beijing, China. The assessment is based on flux measurements, emission modeling, and atmospheric dispersion modeling. The emission rates of NH_3_ and H_2_S were measured with a dynamic flux chamber for seven consecutive days and then detrended to eliminate the impact of the meteorological predictors using a release modification factor *R*_0_. Moreover, the NH_3_ and H_2_S ambient concentrations were predicted using a steady-state Gaussian plume model (AERMOD modeling system) and were used to assess the related non-carcinogenic health risks to residents around the dairy farm.

## Methods

### Dairy farm

**Fig. 1:**
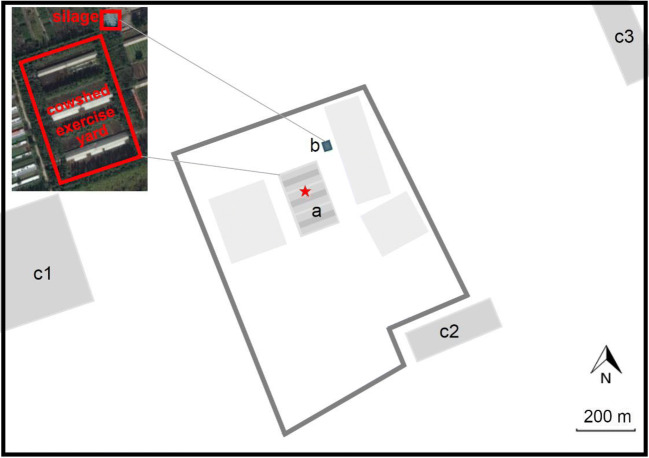
Location and surrounding of the dairy farm. a Feedlot pens (including cowshed and exercise yard); b silage storage; c1 village 1; c2 village 2, c3 village 3; pentagram stands for the sampling site

This study was conducted at a dairy farm in Beijing, China (40.10 N, 116.16 E). The site is located about 30 km north from the center of Beijing and it is surrounded by three villages within a 1 km radius (Fig. 1). The detailed description of the site, the surroundings of the farm, and the topography of the area have been presented in a previous study (Wu et al. 2019). Briefly, the terrain around the farm is mostly flat, and the land is mainly used for farming. The dairy farm has an area of ~ 0.67 km^2^, comprised of feedlot pens, feed mill, slurry treatment workshop, and administrative office. The farm has three feedlot pens with a total area of about 42,000 m^2^. The feedlot pen consists of an exercise yard with a brick floor and a cowshed with a solid concrete floor. The cowshed consists of two specular sections with a manger and a row of free stalls, separated by a central aisle (Fig. 1). About 300 cows are raised in the feedlot pens. The average body mass of the cows is 600 kg or 1.2 AU (1 AU = 500 kg), and the daily milk production per cow is in the range of 27–33 kg cow^−1^ day^−1^. The feedstuff consists of 20 kg corn silage, 11 kg concentrate (maize, bean pulp, bran, etc.), 11 kg of alfalfa, gramineae, cotton seeds, and soy flours for each cow per day. The manure is cleaned from the feeding area using a scraper every day and stored in a vacant cowshed near the feedlot pens. The slurry from the feeding area and the milking parlor is disposed in a slurry treatment workshop. The exhaust gas emitted from the slurry treatment workshop is treated by biofiltration and absorption, so gaseous pollutants were assumed for this process.

### NH_3_ and H_2_S emissions

The NH_3_ and H_2_S emissions, *E*_*N*_ and *E*_*H*_ (in mg s^−1^), respectively, from the dairy farm were determined following two methods: (i) by the measurement of the area-specific emission rate using a flux chamber and (ii) by a release modification model that predicts the release variation through meteorological parameters. In doing so, the emission rate is given as an hourly time series.

The sampling site was located in the middle of the feedlot pen surface of the dairy farm (Fig. 1). Air samples were collected during daylight hours (8:00 am to 7:00 pm local time) in May 2017 for seven consecutive days. The samples were collected with a dynamic flux chamber, which has been widely used in previous studies dealing with the determination of odor emission rates (Gallego et al. 2014; Parker et al. 2013a; Prata et al. 2016). The flux chamber consists of a cylindrical enclosure that is a half-dome with a diameter of 0.4 m and an overall height of 0.25 m. The internal volume and area of the flux chamber were 30 L and 0.12 m^2^, respectively.

To determine the NH_3_ and H_2_S emissions, the flux chamber was placed on the feedlot pen surface with the skirt buried approximately 5 cm deep into the topsoil to ensure a good seal around the base of the chamber. Nitrogen flow was swept through the flux chamber using a perforated plastic y-tube configured as a loop along the interior circumference. The nitrogen flow was controlled by a flow meter. Before each sampling, the flux chamber was equilibrated for 24 min with a flow rate of 5 L min^−1^. This procedure is needed to avoid sampling errors derived from pressure disturbances generated by the disposition of the sampling device. The nitrogen flow rate was adjusted to 2 L min^−1^ when air sampling initiated.

NH_3_ and H_2_S emitted from the covered surface were mixed with the nitrogen and flowed out of the chamber to the impingers. NH_3_ and H_2_S in a known volume of the mixed gas were then trapped bubbling into impingers which contained 10 mL of sulfuric acid and 10 mL of cadmium sulfate solution, respectively. Concentrations of H_2_S and NH_3_ in the absorption solutions were measured by analyzing them with UV/vis spectrophotometer according to the national standard method in China (Wu et al. 2017). Detailed procedures regarding the analysis and relevant quality assurance are similar to those reported in our previous work (Wu et al. 2017). During the seven consecutive sampling days, a total of 42 samples were collected. The meteorological conditions (air temperature, relative humidity, and wind speed at the sampling site with a height of 1.5 m and the air temperature inside the flux chamber) during the sampling campaign were measured with a thermo-anemometer (EXTECH, DK5158 45158, USA).

On the basis of the measurements, the area-specific emission factor *e*_A,meas_ (g a^−1^ m^−2^) was calculated from the mean NH_3_ and H_2_S concentrations, *C*_N_ and *C*_H_ (in mg m^−3^), respectively, the volume flow rate *v* = 0.12 m^3^ h^−1^, and the area *A*_flux_ = 0.12 m^2^ of the flux chamber. Using the available area per animal place of *A* = 140 m^2^ and a body mass of *M* = 1.2 AU, the emission factor was also related to the body mass *e*_M,meas_ (kg a^−1^ AU^−1^) and the animal place *e*_AP,meas_ (kg a^−1^ AP^−1^).

Meteorological conditions modify the release of NH_3_ and H_2_S (the meteorological data on the farm will be shown in the “Results” section). This effect was taken into account by a release modification factor *R*, which was calculated using a regression model developed for a commercial naturally ventilated dairy farm (Hempel et al. 2016). The meteorological predictors of the regression model are air temperature *T* (°C), relative humidity *F* (%), and wind speed *W* (m s^−1^). The annual and diurnal change of emissions is taken into account by the Julian day DOY (day of the year) and the time of the day *t* (h) and by the use of sinusoidal functions. The impact of the wind direction was not considered herein due to the open space of the exercise yard. The hourly release modification factor *R* reads as

$$ \log R={c}_1\kern0.5em \sin \kern0em \left(\frac{2\kern0.5em \pi }{365} DOY\right)+{c}_2\kern0.5em \cos \kern0em \left(\frac{2\kern0.5em \pi }{365} DOY\right)+{c}_3\kern0.5em \sin \kern0em \left(\frac{2\kern0.5em \pi }{24}t\right)+{c}_4\kern0.5em \cos \kern0em \left(\frac{2\kern0.5em \pi }{24}t\right)+{c}_5\kern0.5em T+{c}_6\kern0.5em F+{c}_7\kern0.5em W $$

with the coefficients of the regression model *c*_1_–c_7_ according to Hempel et al. (2016).

To eliminate the bias due to the meteorological parameters during the measurements, reference emission factors *e*_x,0_ were determined. They were related to *x* equal to the area *A*, the animal place AP, and the body mass *M*. These emission factors were calculated by the release modification factor *R*_meas_ to eliminate the impact of the predictors of the emission by *e*_x,0_ = *e*_x,meas_ / *R*_meas_ with the meteorological parameters during the field measurements.

The specific emission factors for the year 2017 *e*_A,17_, *e*_AP,17_, and *e*_M,17_ were calculated using the reference specific emission factors *e*_A,0_, *e*_AP,0_, and *e*_M,0_ and the time series of the hourly release modification factor *R*_17_ using hourly mean values of meteorological parameters (temperature, relative humidity, and wind speed) for 2017 (8760 h).

### Modeled NH_3_ and H_2_S concentrations in ambient air

Based on dispersion calculations, the impact of the dairy farm emissions on air quality, as well as the health risk of these emissions for residents, can be assessed. Here, the AERMOD modeling system was used to predict NH_3_ and H_2_S ambient concentrations. In essence, the modeling system contains three modules: (i) the AERMOD dispersion model itself, (ii) the AERMET meteorological processor, and (iii) the AERMAP terrain processor. Versions 18081 of these modules were herein used. AERMOD is considered an advanced steady-state Gaussian plume model because it integrates atmospheric dispersion based on planetary boundary layer turbulence structure and scaling concepts. It incorporates the Monin–Obukhov similarity theory to estimate the stability of the atmospheric boundary layer in a continuous manner (Cimorelli et al. 2005; EPA 2018; Perry et al. 2005).

The primary inputs to AERMOD were the NH_3_ and H_2_S emissions (on an hourly basis as previously described) and meteorological data. Regarding the latter, the selection of an appropriate meteorological station that best represents the area surrounding the farm has been recently presented in Wu et al. (2019). For brevity, the reader is referred to this work for a complete description of the meteorological input data. In short, the meteorological station Haidian has been selected for the investigation. A 1-year time series of meteorological observations for 2017 from that station has been used.

The modeling protocol followed the current default regulatory options set in the US Guideline on Air Quality Models (EPA 2017). Time series of ambient pollutant concentrations were calculated on a highly resolved nested grid placed in a model domain with a spatial resolution of 5 × 5 km. Receptors were not placed within the dairy farm boundary, meaning that only the emission impact instantly from the farm fence line has been considered. A total of 6297 receptors at 1.5 m above ground level were defined for this receptor grid network. The feedlot pens were treated as an area source at ambient temperature with a release height of 5 cm. A digital elevation model for the model domain was built using the AERMAP terrain processor with terrain data in SRTM1 (resolution of about 30 m). Within the model domain, elevations from near 40 m to no more than 70 m above sea level occur. When processing meteorological data through AERMET, the AERSURFACE utility in its version 13016 was used to specify the surface characteristics (surface roughness length, albedo, and Bowen ratio). Furthermore, the adjusted surface friction velocity technique was considered. This option has the aim of addressing model performance concerns for stably stratified atmospheric boundary layers under low wind speeds (Pandey and Sharan 2019; Qian and Venkatram 2011). Background pollutant concentrations were not considered so that only the dairy farm emissions are reflected in the NH_3_ and H_2_S ambient concentration estimates. The results are judged conservative because deposition (mainly for NH_3_) is treated as negligible and chemical transformations (for NH_3_ and H_2_S) are not accounted for. Different averaging times (hourly, daily, monthly, and annual) were investigated.

### Determination of the health risk due to the NH_3_ and H_2_S exposure

In the case of noxious gases emitted from the dairy farm, inhalation is mainly considered the primary route of human exposure in the present study. Thus, the non-carcinogenic effects of NH_3_ and H_2_S are assessed by combining the people’s inhalation exposure to compounds in the ambient air with toxicological parameters and the methodology recommended by EPA (2009).

The inhalation exposure to compounds was calculated by estimating the annual mean exposure concentration EC for each receptor exposed to pollutants via inhalation. ECs are time-weighted average concentrations which were derived from the predicted annual mean of the ambient concentrations *C*_a_ for NH_3_ and H_2_S and a weighting factor *f*_T_ taking into account the exposure time, as shown in the following equation (EPA 2009):

1 $$ \mathrm{EC}={C}_{\mathrm{a}}\ {f}_{\mathrm{T}} $$

with EC for each compound, and the predicted annual mean concentration *C*_a_ in μg m ^−3^. The weighting factor for the exposure *f*_T_ gives the portion of time for 20 years when exposure can be expected. The weighting factor was calculated for an exposure of 24 h per day, 350 days per year, which gives *f*_T_ = 0.959 (Bari and Kindzierski 2017; Wu et al. 2018). For non-carcinogenic effects, the health risk is expressed by the hazard index HI. Moreover, people are typically exposed through inhalation to a mixture of the gaseous compounds rather than individual pollutants. Thus, a cumulative non-carcinogenic HI was calculated to account for the simultaneous exposure to NH_3_ and H_2_S (EPA 2009; Mustafa et al. 2017; Wu et al. 2018). The HI was calculated by means of the exposure concentration EC and the reference concentration RfC value for chronic inhalation exposure as HI = EC / RfC. The reference concentration for NH_3_ is RfC = 0.002 mg m^−3^; for H_2_S, it is RfC = 0.5 mg m^−3^ (EPA 2009).

## Results

The emission factor of ammonia and hydrogen sulfide was realized by a twofold strategy: (1) the emission factor *e* was first determined directly from the measurements; (2) using the release modification factor *R*, the measurements were detrended to eliminate the impact of the predictors during the measurements, which gives the reference emission factor *e*_0_.

Table 1 summarizes the statistics of the specific emission factor *e*, which is related to the emission area *A* (m^2^), the number of animal places AP (−) in the barn, and the body mass *M* (AU). These three parameters define the activity value. For example, the emission rate *E* can be calculated by the body mass-specific emission rate *e*_M_ and the activity value *M* according to *E = e*_M_ *M*. The reference specific emission factors *e*_0_ were detrended by *R*_0_. The specific emission factors *e*_A_, *e*_AP_, and *e*_M_ are overestimated in relation to the reference specific emission factors *e*_A,0_, *e*_AP,0_, and *e*_M,0_ by a factor of 4.23 for NH_3_ and 8.00 for H_2_S. The overestimation is primarily caused by the fact that the measurements were performed during periods which are characterized by higher emissions. The higher factor for H_2_S can be explained because the measurements were taken only during noon with higher air temperatures. On the other hand, the NH_3_ measurements were also performed during daytime, which results in higher emissions due to higher animal activity.

**Table 1:** Measured specific emission factors and the corresponding reference emission factors

	Measurements			Reference values		
	Area *e*_A,meas_	Animal place *e*_AP,meas_	Body mass *e*_M,meas_	Area *e*_A,0_	Animal place *e*_AP,0_	Body mass *e*_M,0_
**NH**_**3**_	g a^−1^ m^−2^	kg a^−1^ AP^−1^	kg a^−1^ AU^−1^	g a^−1^ m^−2^	kg a^−1^ AP^−1^	kg a^−1^ AU^−1^
Maximum	61.6	8.63	7.19	21.53	3.01	2.51
Minimum	4.2	0.58	0.49	0.80	0.11	0.09
Mean value	18.3	2.56	2.13	5.76	0.81	0.672
**H**_**2**_**S**	mg a^−1^ m^−2^	g a^−1^ AP^−1^	g a^−1^ AU^−1^	mg a^−1^ m^−2^	g a^−1^ AP^−1^	g a^−1^ AU^−1^
Maximum	295.9	41.4	34.5	40.04	5.61	4.67
Minimum	134.5	18.8	15.7	10.11	1.42	1.18
Mean value	213.3	29.9	24.9	24.65	3.45	2.88

Measured specific emission factors (*e*_A,meas_, *e*_AP,meas_, and *e*_M,meas_): related to the emission area *A* (m^2^), the number of animal places AP (−) in the barn, and the body mass *M* (AU). Corresponding reference emission factors (*e*_A,0_, *e*_AP,0_, and *e*_M,0_): detrended by the release modification factor *R*_meas_ for NH_3_ and H_2_S, calculated for the meteorological parameters during the field measurements. Mean value: arithmetic mean value

**Fig. 2:**
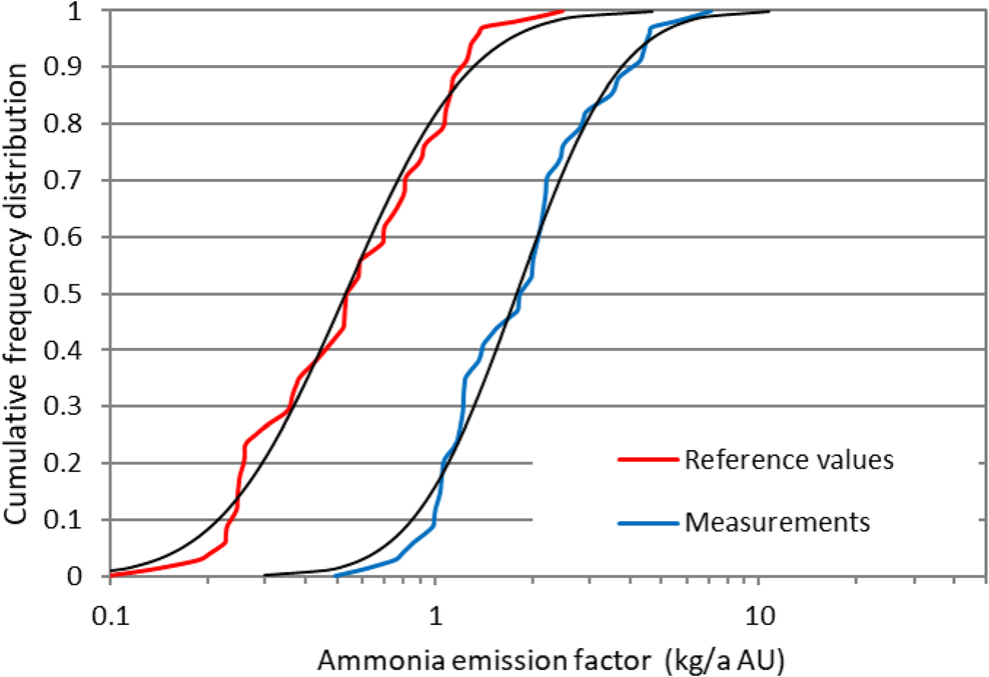
Empirical cumulative distribution function of the measured body mass-specific emission factor *e*_M,meas_ (kg a^−1^ AU^−1^) and the body mass-specific reference emission factor *e*_M,0_ (kg a^−1^ AU^−1^) for the NH_3_ emissions from the dairy farm for the field measurements. Black lines show the theoretical log-normal distributions. The mean value of the measured emission factor *e*_M,meas_ is 2.13 kg a^−1^ AU^−1^; the mean reference emission factor *e*_M,0_ is 0.672 kg a^−1^ AU^−1^ (Table 1)

A log-normal distribution can describe the empirical measurements. Figure 2 shows the body mass-specific emission factor *e*_M,meas_ for the field measurements and the body mass-specific reference emission factor *e*_M,0_ for NH_3_. The corresponding log-normal distributions for the two emission factors were calculated by the mean value and the standard deviation of the logarithmically transformed emission factors *e*_M,meas_ and *e*_M,0_, respectively. The emission factor for H_2_S was not graphically depicted because only 7 measurements are available. The fact that in most studies log-normal distributions of the emission rate are selected can be explained by the multiplicative modulation of the emission by several predictors (Brancher et al. 2020; Limpert and Stahel 2011; Limpert et al. 2001).

Table 2 shows the NH_3_ and H_2_S emission factors for 2017 *e*_A,17_, *e*_AP,17_, and *e*_M,a_, related to the area *A*, the animal place AP, and the body mass *M*. The annual mean values were calculated by hourly meteorological observations of the Haidian station for 2017 (as shown in our previous work (Wu et al. 2019)).

**Table 2:** Statistics of the emission factors for 2017 of NH_3_ and H_2_S

	Emission factors for 2017		
	Area *e*_A,17_	Animal place *e*_AP,17_	Body mass *e*_M,17_
**NH**_**3**_	g a^−1^ m^−2^	kg a^−1^ AP^−1^	kg a^−1^ AU^−1^
Maximum	100.2	14.0	11.7
Minimum	0.11	0.02	0.01
Mean value	8.49	1.19	0.99
**H**_**2**_**S**	mg a^−1^ m^−2^	g a^−1^ AP^−1^	g a^−1^ AU^−1^
Maximum	429.3	60.1	50.1
Minimum	0.49	0.07	0.06
Mean value	36.40	5.10	4.25

The emission factors (*e*_A,17_, *e*_AP,17_, and *e*_M,17_, which were related to the emission area *A*, the animal place AP, and the body mass *M.*) were calculated based on the reference specific emission factors *e*_A,0_, *e*_AP,0_, and *e*_M,0_ and the release modification factor *R*_17_ for the year 2017. Mean value is the arithmetic mean

The discrepancy between the annual mean values *e*_x,17_ (Table 2) compared with the emission factors of the measuring period *e*_x,meas_ (Table 1) is caused by differences in the meteorological conditions, which are expressed by the release modification factor *R*. The air samples were only collected in May during daytime. Table 3 summarizes the differences between the meteorological parameters. The overestimation of *e*_x,meas_ was caused by the higher temperatures during the measurements in May compared with the annual mean temperature and due to the fact that the measurements were conducted during daytime with higher animal activity.

**Table 3:** Maximum, minimum, and mean values of the meteorological predictors used to calculate the emission factors both for the measuring period and for the year 2017

	Temperature *T* (°C)		Relative humidity *F* (%)		Wind speed *W* (m s^−1^)	
	Measuring period	Annual mean	Measuring period	Annual mean	Measuring period	Annual mean
Maximum	36.9	38.7	63	97	6.5	6.8
Minimum	19.5	− 10.8	12	5	0.4	0.5
Mean	28.0	11.4	30	42	3.1	1.7

**Fig. 3:**
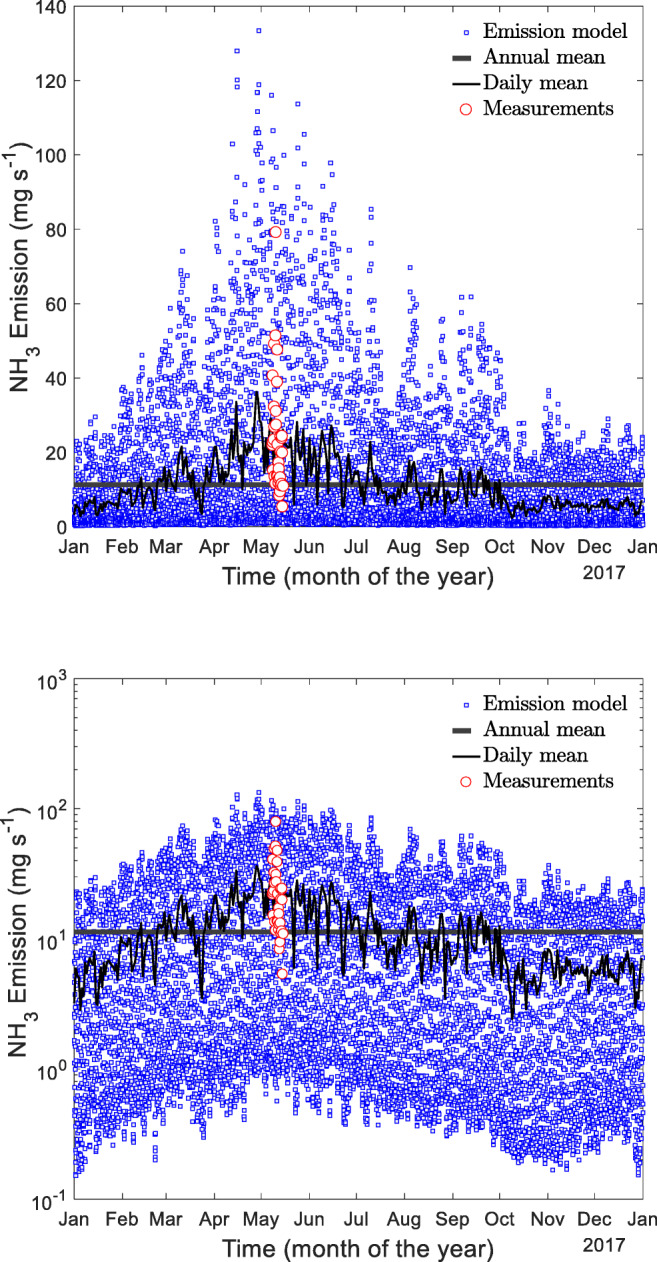
Time course of the ammonia emission rate *E* (mg s^−1^) calculated by the body mass-specific emission factor *e*_M,0_ = 0.672 kg a^−1^ AU^−1^ and the total body mass inside the barn of *M* = 360 AU, calculated for the 2017 meteorological dataset of the Haidian station by the release modification factor *R*_17_. The field measurements are shown in red. The annual mean value is 11.3 mg s^−1^ shown by the black line. Panel A is linearly, panel B logarithmically diagramed

The hourly time series of NH_3_ emissions for 2017 shows a substantial variation over the year (Fig. 3). As previously mentioned, the air samples were collected during the warm season (May and only during daytime) which causes an overestimation of the emissions. This overestimation was eliminated by the use of the release modification factor for the period of the field measurements. A daily mean was also computed from the hourly values and overlaid in Fig. 3 to depict the annual daily pattern of the NH_3_ emissions.

**Fig. 4:**
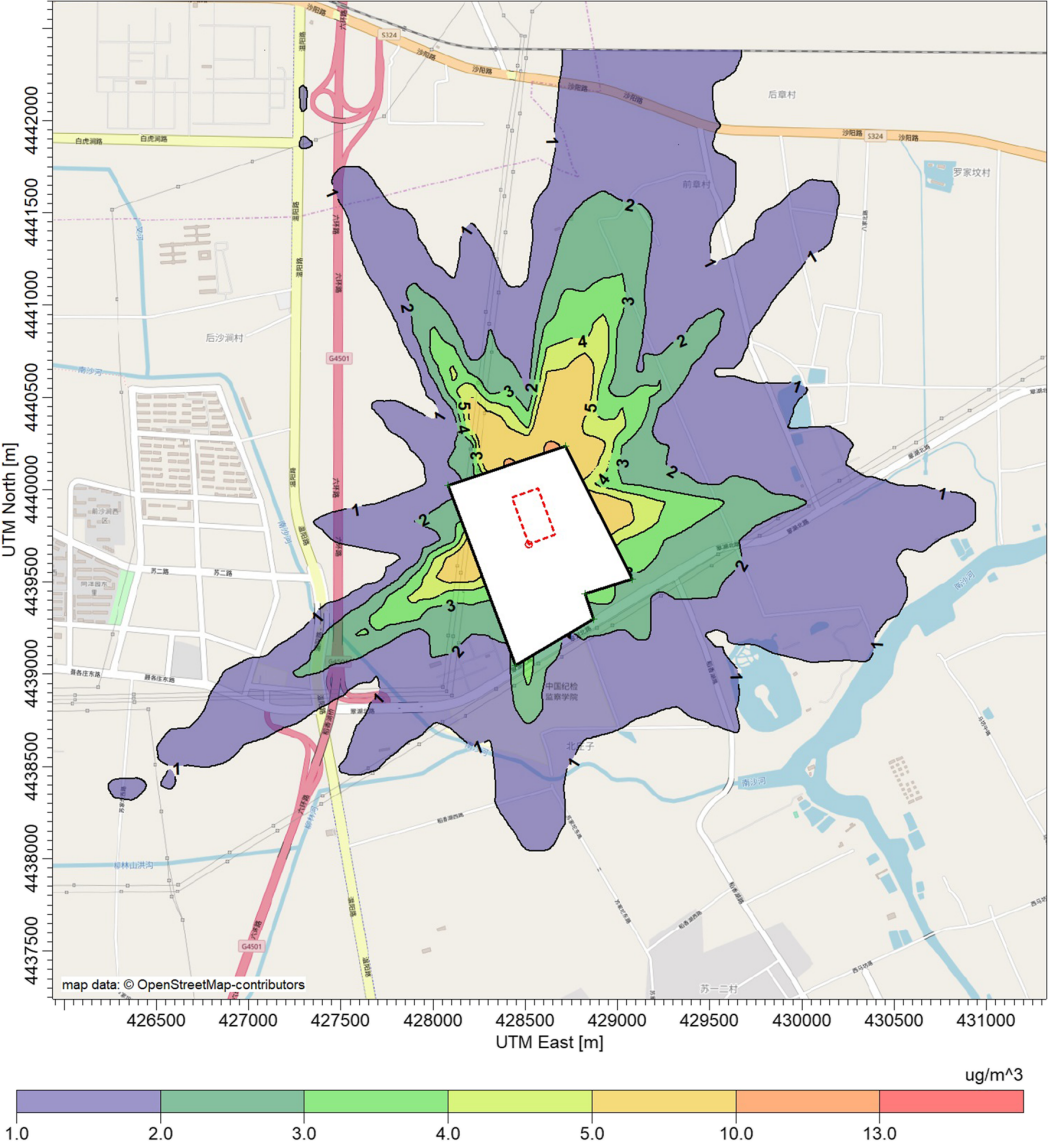
Contour plot of hourly mean concentrations of ammonia in the surrounding area of the dairy farm. The black circle stands for the fence line of the farm, and the red rectangle stands for the feedlot pen areas

Figure 4 shows the contour lines of first high hourly mean concentrations of NH_3_ around the dairy farm. Typically, the elongation of the contour lines tended to be greater in the prevailing winds, mainly driven by the frequency of wind directions. The maximum hourly concentrations for NH_3_ and H_2_S were 13.2 μg m^−3^ and 0.056 μg m^−3^, respectively. The maximum distances for a NH_3_ concentration of 5 μg m^−3^ is about 350 m towards north-northeast (NNE) and about 250 m towards southwest (SW). These maximum distances reach about 1300 m and 1200 m, respectively, for the concentration of 2 μg m^−3^. For lower NH_3_ concentration of 1 μg m^−3^, the separation distances nearly doubled compared with that of 2 μg m^−3^, forming a larger encompassed area (the purple area in Fig. 4). Moreover, the concentration of NH_3_ at the fence line is in the range of 2 μg m^−3^ (NNE)–10 μg m^−3^ (SW). These values are 2–3 orders of magnitude lower than the current emission standards in China (1.5 mg m^−3^) (GB14554 1993), and also far below the upcoming new version of the emission standards (0.2 mg m^−3^).

**Fig. 5:**
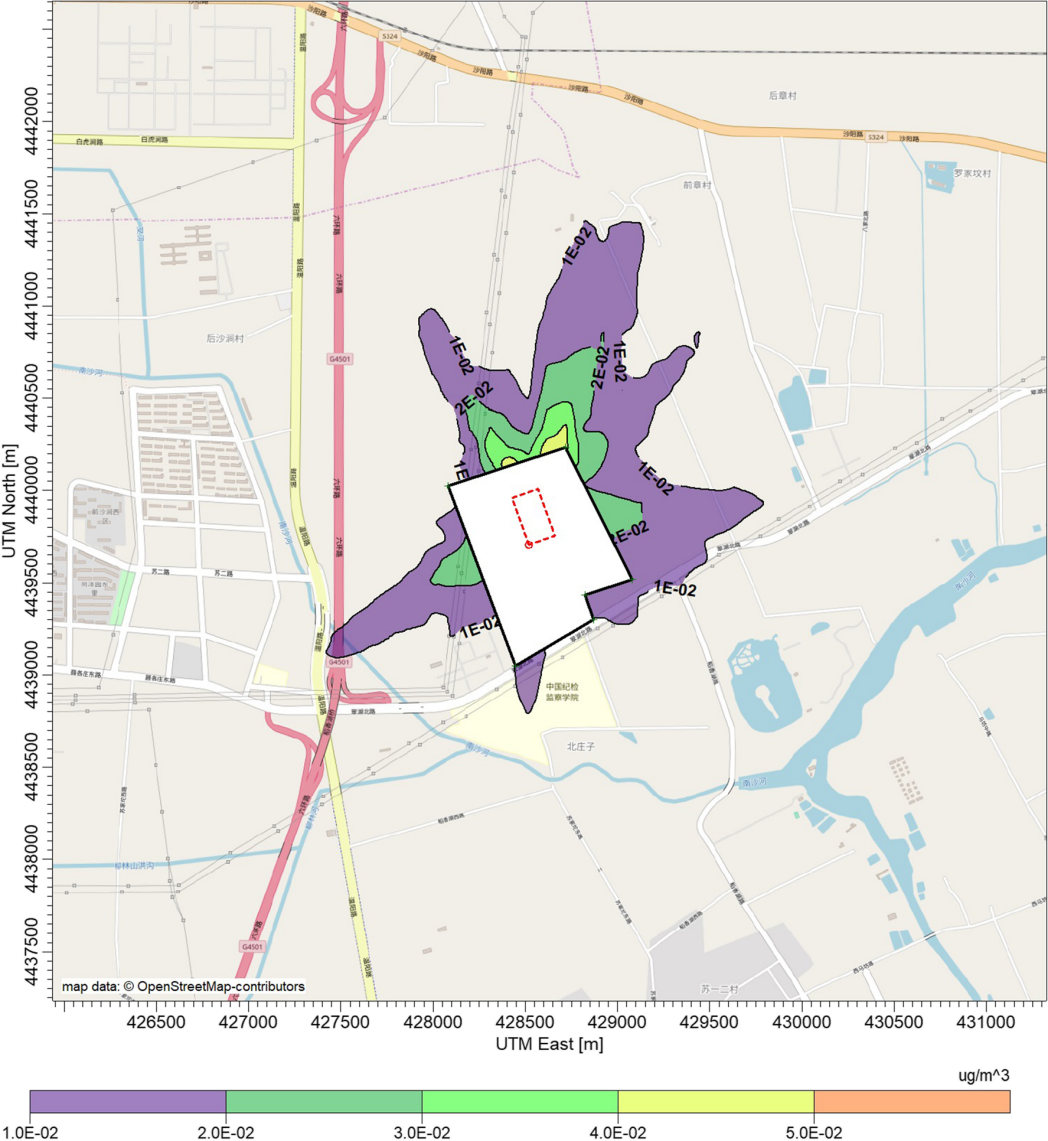
Contour plot of hourly mean concentrations of hydrogen sulfide in the surrounding area of the dairy farm. The black circle stands for the fence line of the farm, and the red rectangle stands for the feedlot pen areas

On the other hand, the contour lines of the first high hourly mean ambient concentrations of H_2_S are shown in Fig. 5. From a visual inspection of this figure, the shape of the encompassed area of H_2_S is comparable with those of NH_3_. This is due to the fact that NH_3_ and H_2_S levels around the dairy farm shared the same origin and dispersion pathway. The maximum distance for an H_2_S concentration of 0.04 μg m^−3^ is about 150 m towards NNE. For a lower H_2_S concentration of 0.02 μg m^−3^, the maximum separation distances are about 700 m towards NNE and 400 m towards SW. The concentration of H_2_S at the fence line ranges from 0.02 μg m^−3^ to 0.04 μg m^−3^, which is three orders of magnitude lower than the current and the upcoming emission standards in China (0.06 mg m^−3^ and 0.02 mg m^−3^, respectively) (GB14554 1993).

For the other averaging times investigated in this work (daily, monthly, and annual), it was observed that the impact of NH_3_ and H_2_S emissions was even lower, so that the creation of contour maps was unnecessary.

**Fig. 6:**
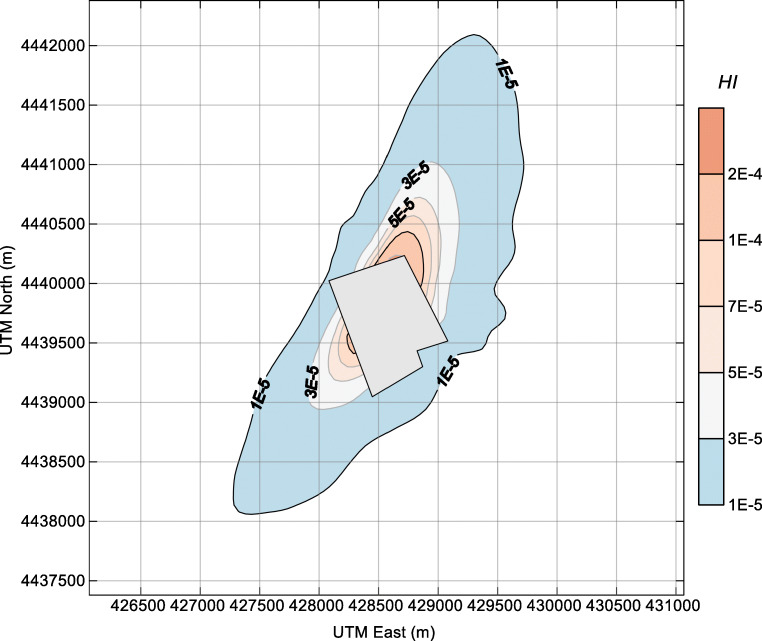
Contour plot of the criterion for non-carcinogenic risk HI of ammonia in the surrounding area of the dairy farm. The farm fence line is depicted in the center of the plot

**Fig. 7:**
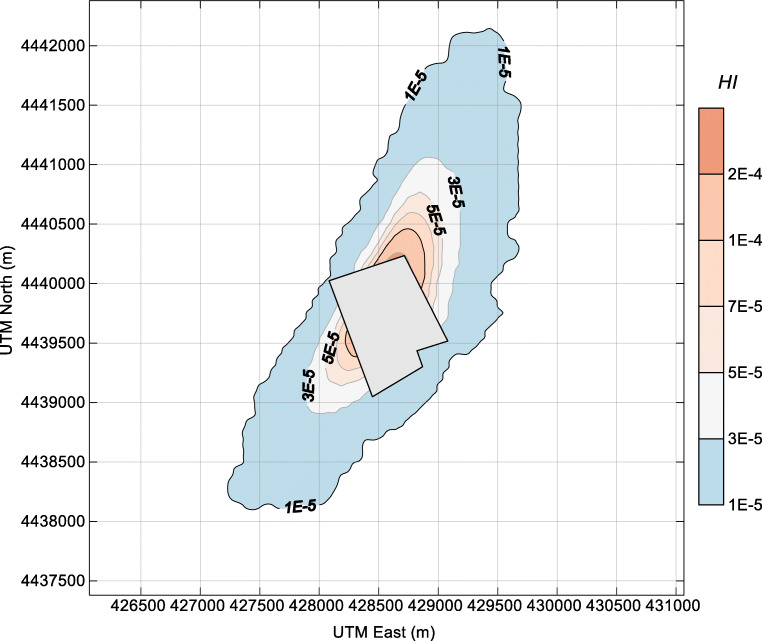
Contour plot of the criterion for non-carcinogenic risk HI of hydrogen sulfide in the surrounding area of the dairy farm. The farm fence line is depicted in the center of the plot

**Fig. 8:**
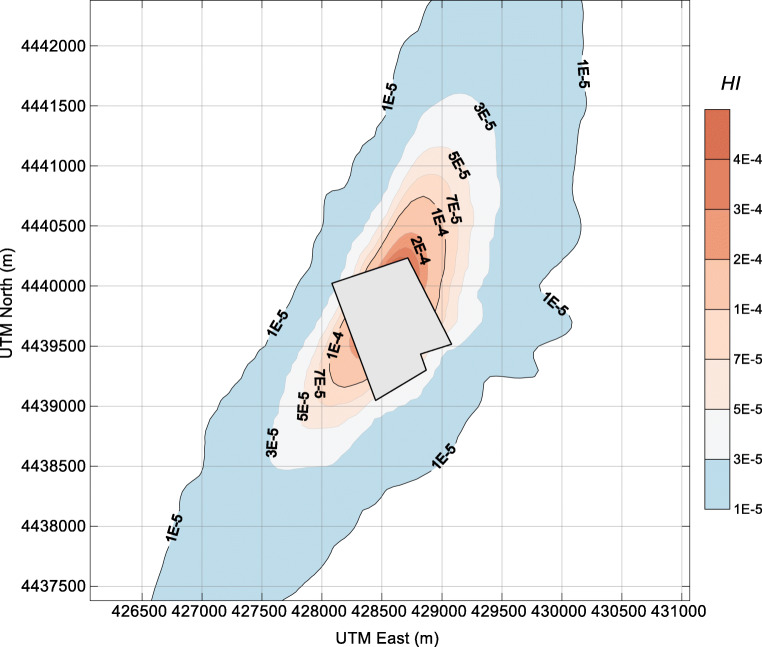
Contour plot of the criterion for cumulative carcinogenic risk (*∑*HI) of ammonia and hydrogen sulfide in the surrounding area of the dairy farm. The farm fence line is depicted in the center of the plot

From a toxicological point of view, the NH_3_ and H_2_S exposure, described by ambient concentrations around the dairy farm, could pose potential health risks to nearby residents. Hence, the non-carcinogenic risks (quantified by HI) of NH_3_ and H_2_S were assessed according to the methodology recommended by the USEPA (EPA 2009). For this purpose, we considered the annual mean concentration calculated by the AERMOD dispersion model (Figs. 4 and 5). The outcomes are displayed in Figs. 6 and 7. Moreover, the cumulative non-carcinogenic risk (quantified by *∑*HI) was calculated to account for the simultaneous exposure to both NH_3_ and H_2_S (Fig. 8). The contour lines of health risks of NH_3_, H_2_S, and *∑*HI stretch from south-southwest (SSW) to NNE. The shape of the contour lines is similar and is mainly driven by the distribution of wind directions.

In general, the HI of NH_3_ and H_2_S around the dairy farm is quite low. The HI of H_2_S is very close to that of NH_3_, although the ambient concentration of H_2_S was far lower than that of NH_3_. The health-related impact posed by H_2_S deserves more attention in similar facilities since the reference concentration (RfC) of H_2_S (0.002 mg m^−3^) is 250 times lower than that of NH_3_ (0.5 mg m^−3^) (EPA 2009). From the perspective of cumulative risk, the *∑*HI values near the fence line of the dairy farm were in the range of 3E-5–4E-4. With the health risk criterion of HI = 1E-4, the maximum separation distances for the NNE and SSW wind directions are approximately 1000 m and 700 m, respectively. For residents who are living in the three surrounding villages (villages 1–3, shown in Fig. 1), the cumulative carcinogenic risks are 3E-5, 1E-5, and 1E-5, respectively. These HI values were 4 orders of magnitude lower than the acceptable risk levels (HI = 1), indicating that the health risk from the ambient NH_3_ and H_2_S around the dairy farm is low.

## Discussion

Emissions from livestock facilities that keep animals both inside barns or in open space are commonly estimated by emission factors and the related activity values. The emission factors are mostly associated with the area which is available for the animals, animal place, or body mass, expressed in AU (1 AU = 500 kg). For dairy farms, the last two factors are closely related because the body mass of a cow is more or less 600 kg or 1.2 AU. The available area per animal shows much more variability, depending on the livestock keeping system. For cubicles, about 10 m^2^ is available for one animal (Baldini et al. 2016). Contrary, in our case, the area per animal is about 140 m^2^. For such a widespread range, the body mass-related emission factor or the animal place-related emission factor seems to be more appropriate for dairy farms.

It is well known that the emission of NH_3_ and H_2_S of livestock farming is influenced by several predictors. The most important ambient predictors are air temperature, wind speed above the release surface, and relative humidity (Maasikmets et al. 2015; Schauberger et al. 2013). Other predictors are the time of the day as a proxy for animal activity. Lonsdale et al. (2017) reported that NH_3_ emissions from livestock feedlots show a strong diurnal cycle, peaking at midday. Yang et al. (2016) and Mukhtar et al. (2008) observed apparent seasonal variations in NH_3_ emissions from dairy feedlots. Joo et al. (2015) concluded that the NH_3_ emissions from dairy barns correlated fairly well with temperature (*R*^2^ = 0.29 to 0.51), but correlated moderately with wind speed (*R*^2^ = 0.01 to 0.46). Feilberg et al. (2017) reported that the concentration of NH_3_ in a cattle farm in summer is 1.9 times higher than that in winter, while for H_2_S, the discrepancy could be 14.6 times since the production of H_2_S from, e.g., sulfate reduction is strongly reduced at low temperatures. Multiple linear regression models built by Wu et al. (2012) showed that wind speed (*P* < 0.001) and air temperature (*P* < 0.001) influence ammonia emissions from dairy cattle buildings significantly. In the current study, a positive monocausal correlation trend was found between NH_3_ emission factors and both the ambient air temperature at the height of 1.5 m and the air temperature inside the flux chamber (*r* = 0.59 and 0.60, respectively, *p* < 0.001, *n* = 35). Based on continuous measurements of ammonia, it has been shown that indoor temperature, air velocity, and animal activity are the most relevant influencing factors that affect ammonia emission (Arogo et al. 2003; Blanes-Vidal et al. 2008; Flesch et al. 2009; Hayes et al. 2006; Huang and Guo 2017; Schauberger et al. 2013; Ye et al. 2011).

The correlation between relative humidity and the NH_3_ emission rate in dairy farms is seen controversial. Adviento-Borbe et al. (2010) reported a poor correlation of *r* = − 0.025 in a free-stall cow barn. Hempel et al. (2016) and Saha et al. (2014) addressed the significant effect of relative humidity on the NH_3_ emissions from a naturally ventilated dairy barn. The cause is complicated and needs further investigation. Ammonia is water-soluble and could be transformed into ammonium (NH_4_^+^) in humid air and thus affect the measurement of gaseous ammonia with infrared photo-acoustic analyzer (Saha et al. 2014). Hadlocon et al. (2014) concluded that high moisture content in the air might also cause cross-interference in the readings of the instrument, and the recommended acid trap method is as a reference for this condition. For confined livestock buildings and naturally ventilated barns, these parameters depend on the indoor climate, which can be simulated by appropriate models (Mikovits et al. 2019; Schauberger et al. 2000).

The impact of these predictors can be analyzed in two ways: (1) by a statistical approach, the impact is examined by regression models (e.g., Hempel et al. 2016; Saha et al. 2013). The major disadvantage of regression models is the limitation of the interpretation of the calculated regression coefficients; (2) by physical-orientated models, which open the opportunity to compare the regression model outputs with other investigations.

In this work, a regression model was applied, which has been developed for a commercial naturally ventilated dairy barn (Hempel et al. 2016) to eliminate the impact of these predictors. Due to comparable geometry and the use for dairy cows, this model seems appropriate. The regression model is based on multilinear regression analysis for air temperature, relative humidity, and wind speed as meteorological predictors, and the day of the year DOY and the time of the day *t* as predictors for animal activity. The limitations of a regression model can be seen by the model of Hempel et al. (2016). This regression model uses the day of the year as a predictor. This parameter shows a strong cross-correlation to air temperature. The impact of air temperature can be seen in detail in Jeppsson (2002), Ni (1998), and Smits et al. (1995) for the NH_3_ release. For the air velocity above the release surface, Ni (1999) showed the importance for the convective mass transfer, which can be parameterized by a power function. A detailed discussion of these predictors can be found in Schauberger et al. (2013). Nevertheless, we used the regression model approach of Hempel et al. (2016) which was developed for a dairy barn in a comparable configuration (364 cows with 70 m^3^ per animal) with an investigated barn.

A major restriction of the applicability of the regression model is the fact that the model was developed for NH_3_ and not for H_2_S. Under the assumption that many gaseous emissions from livestock show a similar behavior concerning the predictors, we decided to use this model also for the H_2_S release as a first educated guess. Alternatively, the emission factors *e* shown in Table 1 could be used as annual mean values without any modifications related to meteorological parameters and the time of the day and the time of the year.

The annual mean emission factors for 2017 of NH_3_ and H_2_S, based on the field measurements and the release modification factor *R*, are summarized in Table 2. The annual mean emission factor of H_2_S (4.24 g a^−1^ AU^−1^) was about 200 times lower than that of NH_3_. Also in Trabue et al. (2011), NH_3_ was found to be 2–3 orders of magnitude higher than other gaseous compounds such as trimethylamine, volatile fatty acid, and phenol emitted from a cattle farm. The overall mean emission factor calculated for the tie housing cow building was 0.99 kg a^−1^ AU^−1^ for NH_3_ in 2017. This value is comparable with the emission rates of dairy farms located in some other countries. Misselbrook et al. (2001) reported that the NH_3_ emission factor from a dairy cow collecting yard is 1.2 kg a^−1^ AU^−1^ based on concentration measurements with dynamic chambers in England. Also, Mukhtar et al. (2009) reported that NH_3_ emission factors from free-stall barns and open-lots of a free-stall dairy in central Texas were 1.25 kg a^−1^ AU^−1^ and 1.00 kg a^−1^ AU^−1^, respectively. Baldini et al. (2016) summarized that the NH_3_ emission factors on a feeding area varied from 0.62 kg a^−1^ AU^−1^ (concrete floor) to 2.76 kg a^−1^ AU^−1^ (rubber mat) in dairy farms in Italy. Some researchers (Flesch et al. 2007; Leytem et al. 2011; McGinn et al. 2016) reported high NH_3_ emission factors for dairy farms (up to 31.0–54.8 kg a^−1^ per animal), which were several times higher than the annual mean emission factor given in the present study. The discrepancy in NH_3_ emission factors might be caused by dietary differences (Leytem et al. 2011; Maasikmets et al. 2015), performance levels, animal activity (loose or cubicle livestock keeping), manure management (scrapper, slatted floor (Philippe et al. 2007a; Philippe et al. 2007b)), indoor climate (temperature, air velocity above the release surface (Schauberger et al. 2013)), and the configuration and operation of the measuring equipment such as flux chambers and wind tunnels (Parker et al. 2013a). For example, direct measurement methods such as the flux chamber may have a limitation on the spatial variability of observations, especially for large area sources. In this case, diffusion models could be used to calculate the concentrations for the validation of the emission rates (Maasikmets et al. 2015).

The release modification factor *R* of regression models can be used as follows:

1. To avoid a bias which is caused by the environmental conditions during the measuring campaign;
2. To model a time series on the basis of the predictors.

For the first case, the measurements in May showed an overestimation for NH_3_ and H_2_S by a factor of 4.23 and 8.00, respectively. For odor emissions from fattening pigs, a factor of 2 (Schauberger et al. 2013) was assessed. To avoid such a bias by the predictors of the release modification factor (meteorological parameters, time of the day, and time of the year), continuous measurements over an entire year have to be undertaken. The result of such time series calculated by the release modification factor has been shown in Fig. 2.

The emission factors were related to the area of the barn, the number of animal places, and the body mass. These are the activity values which are predominantly used to scale the emission to calculate the pollutant emission rate. For a dairy barn with a constant body mass, all activity values can be used. For other livestock keeping systems (e.g., broiler, fattening pigs), the animal growth has to be taken into account.

The biological relevance and the toxicity of NH_3_ and H_2_S are well known. In general, the health impact is described by a non-linear dose–response function (Hilderman and Wilson 1999; ten Berge et al. 1986). The health-related exposure *H* can be calculated by a time series of the ambient concentration *C* and the exposure time *T* according to *H* ∝ *C*^ *α*^*T* with an exponent *α* (Miller et al. 2000) between 1.0 and 3.5 (ten Berge et al. 1986). For the health impact of NH_3_ and H_2_S, the exponent is assumed as *α* = 1. This assumption results in a linear relationship as proposed by EPA (2009). The major benefit of this approach relies on the fact that the annual mean value can be used for health assessments instead of a time series of ambient concentrations, which would be necessary for a non-linear approach (*α* > 1). This means that dispersion model calculations are much more robust for the annual mean compared with the non-linear dose–response relationship.

In the present study, the non-carcinogenic health risk of H_2_S and NH_3_ around the dairy farm is very low, practically negligible in view of the methodologies and the acceptable risk levels herein considered. It might be the case that the assessment of individual substances does not show the actual extent of the risk of olfactory annoyances to the population. In our previous study (Wu et al. 2019), the assessment of the odor annoyance from the same dairy farm confirmed a considerable impact.

The cumulative non-carcinogenic risk was herein calculated by the sum of individual HI of H_2_S and NH_3_. This sum generally yields an estimated hazard index for multiple chemicals assessed via a hazard-based approach, and it is valid only for toxics that affect the same target organ or organ system. However, if the individual HI is greater than 1, it is generally more appropriate to derive separate HI for each target organ of concern (EPA 2009). For the calculation of non-carcinogenic health risk, the parameters (20 years, 350 days, and 24 h exposure time) represent a specific type of resident according to the methodology recommended by the EPA (2009). That is, those individuals live and work in the surroundings over that period. Other groups of residents, which are likely to be less exposed to the ambient pollutant concentrations from the farm, have not been taken into account here. For example, a resident that lives in the region but studies/works away from the source or a resident that works near the source but resides away from it is not taken into account. Farm workers, which on the other hand are likely to be exposed to higher concentrations, have not been considered either. An example of a risk assessment study on human exposure to H_2_S concentrations near two wastewater treatment plants in Curitiba, Brazil, that has considered different groups, can be found elsewhere (Godoi et al. 2018). Overall, the contour lines show a similar shape as the wind rose, which indicates that the nature of health-related exposure is intimately connected to the meteorology of a particular site (Brancher et al. (2019).

## Conclusions

The emissions as well as the health risk of NH_3_ and H_2_S in the context of a dairy farm located in Beijing, China, were evaluated. The results of the flux chamber measurements showed that the emission factors of NH_3_ were much higher than those of H_2_S. The encompassed area of the contour lines of the ambient concentrations of NH_3_ was much larger than that of H_2_S, while the contour lines of the health risk of these pollutants are similar to each other. Overall, the results suggest that health risks due to ambient concentrations of NH_3_ and H_2_S are unlikely to exist for the population living near the dairy farm.

## Data Availability

The datasets used in the current study are available from the corresponding authors on reasonable request.
